# A Novel Approach to Combinatorial Problems: Binary Growth Optimizer Algorithm

**DOI:** 10.3390/biomimetics9050283

**Published:** 2024-05-09

**Authors:** Dante Leiva, Benjamín Ramos-Tapia, Broderick Crawford, Ricardo Soto, Felipe Cisternas-Caneo

**Affiliations:** Escuela de Ingeniería Informática, Pontificia Universidad Católica de Valparaíso, Avenida Brasil 2241, Valparaíso 2362807, Chile; leivadante14@gmail.com (D.L.); benjamin.alejandro.ramos.tapia@gmail.com (B.R.-T.); ricardo.soto@pucv.cl (R.S.); felipe.cisternas.c@mail.pucv.cl (F.C.-C.)

**Keywords:** set-covering problem, metaheuristics, optimization, combinatorial problems

## Abstract

The set-covering problem aims to find the smallest possible set of subsets that cover all the elements of a larger set. The difficulty of solving the set-covering problem increases as the number of elements and sets grows, making it a complex problem for which traditional integer programming solutions may become inefficient in real-life instances. Given this complexity, various metaheuristics have been successfully applied to solve the set-covering problem and related issues. This study introduces, implements, and analyzes a novel metaheuristic inspired by the well-established Growth Optimizer algorithm. Drawing insights from human behavioral patterns, this approach has shown promise in optimizing complex problems in continuous domains, where experimental results demonstrate the effectiveness and competitiveness of the metaheuristic compared to other strategies. The Growth Optimizer algorithm is modified and adapted to the realm of binary optimization for solving the set-covering problem, resulting in the creation of the Binary Growth Optimizer algorithm. Upon the implementation and analysis of its outcomes, the findings illustrate its capability to achieve competitive and efficient solutions in terms of resolution time and result quality.

## 1. Introduction

There are a series of real-world problems that exhibit high complexity [1,2,3,4,5]. These are characterized by the presence of multiple local optima and a global optimum that, due to the high number of variables and current computing capabilities, would take unimaginable amounts of time to solve the mathematical functions that model these problems. Therefore, techniques have been studied and developed to efficiently obtain optimal solutions, with metaheuristic algorithms being among the most popular. These algorithms stand out mainly because of their flexibility, their ease of implementation, and the lack of a need for gradients when solving a problem.

The set-covering problem is an optimization challenge in the fields of computer theory and operations research. In this problem, the goal is to find the most efficient way to cover a set of elements using a smaller set of subsets, where each subset has an associated cost. The objective is to minimize the total cost by selecting an appropriate set of subsets so that all elements are covered at least once. The set-covering problem has applications in various fields, such as route planning, resource allocation, and general decision-making. Solving this problem involves striking a balance between the number of selected subsets and the total cost required to achieve complete coverage [6].

One of the most relevant characteristics that a metaheuristic should have is the ability to possess operators that allow both exploration and exploitation of the search space. Exploitation refers to the algorithm’s ability to perform a local search, while exploration refers to its ability to perform global searches, thus enabling the heuristic to find optima throughout the entire search space.

The main aims of our work are the following:

- Implement a binary version for Growth Optimizer.
- Solve the set-covering problem with our proposal.
- Carry out a deep analysis of the behavior of our proposal. We will analyze the convergence, execution times, best results obtained, distribution of fitness in the independent runs executed, and balance of exploration and exploitation.

The present work is organized as follows. Section 2 defines the combinatorial optimization problems. Section 3 describes the concept of metaheuristics. Section 4 describes the theoretical background and the set-covering problem along with its mathematical model. Section 5 defines the Growth Optimizer algorithm and the mathematical model, and the used operators are presented. Section 6 presents our proposal: a Binary Growth Optimizer for solving the SCP. In Section 7, an analysis and a discussion of the obtained results are carried out. Finally, Section 8 provides the conclusions and future work.

## 2. Combinatorial Optimization Problems

Optimization problems are a type of problem in mathematics and computer science that involve finding the best solution from a set of possible solutions, according to certain criteria or constraints. In these problems, the objective is to maximize or minimize an objective function, which usually represents a measure of quality or efficiency [7].

One of the major challenges in combinatorial optimization is the current computing capacity, which often cannot deliver optimal solutions efficiently. Among the most famous problems is the Traveling Salesman Problem [5], where individuals must decide the shortest route to visit n cities to optimize their fuel costs. The number of possibilities to calculate to solve this problem is equal to “*n*” factorial, meaning that the number of possible solutions grows factorially with the number of cities. Using the most powerful computer available today to solve a problem like this for 20 cities would require approximately 15,000 years to provide the optimal solution. In 1954, techniques such as linear programming, heuristics, and branch and bound were first used to solve instances of the Traveling Salesman Problem. These techniques have been the most successful methods for solving these types of problems to this day.

In order to classify combinatorial optimization problems by complexity, various research efforts have led to four basic categorizations: P-type, NP-type, NP-complete, and NP-hard. P-type problems are those that can be solved in polynomial time “*n*” by deterministic algorithms, where “*n*” is the problem size. NP-type problems can be solved in polynomial time of the same degree as the problem size “*n*” by nondeterministic algorithms. NP-complete problems are at least as difficult as NP problems but are considered the most challenging within this classification. They can be solved in polynomial time through a polynomial reduction. Lastly, NP-hard problems are at least as difficult as NP problems, but no algorithm is known that can solve them in polynomial time. It is important to note that all NP-complete problems are within the NP-hard classification, but not all NP-hard problems fall under the NP-complete category [8].

### 2.1. Continuous Optimization Problems

Continuous optimization problems are those in which the goal is to minimize an objective function within a search space defined by continuous boundaries or constraints. In these problems, solutions are continuous values, and the objective function can be computationally expensive to evaluate. There is no requirement for discrete data structures, and the search space is continuous [9].

### 2.2. Discrete Optimization Problems

Discrete optimization problems are characterized by having solutions represented as discrete data structures rather than continuous values. These data structures can include ordinal, categorical, or binary variables, permutations, strings, trees, or other discrete representations. In discrete optimization problems, the continuous boundaries or constraints are often not necessary. These problems involve finding the best combination or configuration of elements within a discrete set of options [9].

## 3. Metaheuristics

Metaheuristics, also known as stochastic search algorithms, are characterized by an iterative search that uses stochastic procedures to generate the next iterations. The next iteration may contain a candidate solution to be the best local optimum.

These algorithms are considered robust and easy to implement because they do not rely on structural information from an objective function, such as gradient information or convexity. This feature has contributed to their popularity in the field of combinatorial optimization. However, many of these algorithms require specifying various configuration parameters, such as population sizes, variation operators, or distribution functions, making it necessary to fine-tune them to solve different problems.

The most basic metaheuristics are instance-based. These algorithms maintain a single solution or a population of candidate solutions. The construction of new candidate solutions depends explicitly on the solutions generated previously. Prominent examples of representatives in this category include simulated annealing [10], evolutionary algorithms (EAs) [11], and tabu search [12]. To delve deeper into metaheuristics, the book by El-Ghazali Talbi is recommended [13].

## 4. The Set-Covering Problem (SCP)

The SCP is a classical optimization problem defined by a binary matrix, denoted as A. In this matrix, each cell is represented as a binary value, where $a_{ij}\in \left[0,1\right]$, and *i* and *j* are the size of m-rows and n-columns, respectively:

$$\left[\begin{array}{cccc} a_{11} & a_{12} & \ldots & a_{1n}\\ a_{21} & a_{22} & \ldots & a_{2n}\\ \vdots & \vdots & \ldots & \vdots \\ a_{m1} & a_{m2} & \ldots & a_{mn} \end{array}\right]$$

Let i∈1,2,⋯,m and j∈1,2,⋯,n represent the sets of rows and columns, respectively. The primary objective of the problem is to minimize the cost associated with the subset S⊆J, subject to the constraint that all rows i∈I must be covered by at least one column j∈J. It is important to note that the inclusion of column *j* in the subset of solution S is represented as 1, and 0 otherwise. The problem at hand can be formally defined as the set-covering problem (SCP), which seeks to

(1) $$\mathrm{Minimize}\;Z=\sum_{j\in J}c_{j}\cdot x_{j}$$

Subject to Equations (2) and (3),

(2) $$\begin{array}{cc} \sum_{j\in J}a_{ij}\cdot x_{j} & \geq 1\;\forall i\in I\;(\mathrm{each}\;\mathrm{row}\;\mathrm{must}\;\mathrm{be}\;\mathrm{covered}) \end{array}$$

(3) $$\begin{array}{cc} x_{j} & \in \{0,1\}\;\forall j\in J\;(\mathrm{binary}\;\mathrm{variables}) \end{array}$$

### 4.1. Applications

The SCP has a variety of real-world applications, making it highly relevant in the optimization field. These real-world problems often pose a significant computational burden on teams, necessitating the development of techniques to obtain feasible solutions within a reasonable timeframe. Examples of these real-world applications include the following.

#### 4.1.1. Organization of the Serbian Postal Network

Efficiently organizing a postal network that ensures global service coverage is a challenging task. It involves the strategic placement of physical stations or access points where local residents can send parcels or deposit items. This problem is subject to additional constraints related to population density, access point placement costs, and city size. The primary objective is to minimize the number of permanent postal units within the postal network. This optimization process reduces the operational costs for the postal operator and minimizes the total number of employees involved in the service [14].

#### 4.1.2. Sibling Relationship Reconstruction

In the field of genetics, there is a challenge in modeling and reconstructing sibling relationships among individuals of a single generation when parental genetic information is unavailable. This problem holds significant importance, as knowledge of familial relationships is crucial in biological applications, such as studies of mating systems, the population management of endangered species, or the estimation of hereditary traits [15].

#### 4.1.3. Periodic Vehicle Routing Problem

In this context, the problem involves determining the minimum-cost routes for each day within a given planning horizon. These routes come with constraints that require each customer to be visited a specified number of times (chosen from a set of valid day combinations) and ensure that the required quantity of products is delivered during each visit. Another critical constraint is that the number of daily routes (each respecting the vehicle’s capacity) should not exceed the total number of available vehicles [16].

### 4.2. Solving Set-Covering Problem Review

The set-covering problem seeks a subset of decision variables that satisfy a minimum cost, and Crawford et al. proposed an improved binary monkey search algorithm (MSA) to handle the SCP [6]. The algorithm employs a novel climbing process to enhance exploration capability and a new cooperative evolution to reduce the number of infeasible solutions. Jaszkiewicz compared the computational efficiency of three state-of-the-art multi-objective metaheuristic algorithms in the SCP [17], and computational effort was compared in achieving the same solution quality by calculating average of scalarizing functions in representative samples. Kılıç and Yüzgeç proposed an antlion optimization (ALO) algorithm for the quadratic assignment problem based on contest selection [18]. In the random walking process of ALO, a tournament selection strategy is introduced to replace the roulette method, and several equations in ALO are modified.

The minimum labeling spanning tree (MLST) is an NP-hard problem commonly applied in communication networks and data compression. To address this problem, Lin et al. introduced a binary FA that repairs infeasible solutions and eliminates redundant tags [19], and the algorithm is more suitable for discrete optimization. Vehicular ad hoc networks (VANETs) require robust paths connecting all nodes to achieve reliable and efficient information transmission, but classic graph theory only yields a minimum spanning tree (MST). Zhang and Zhang proposed a binary-coded ABC algorithm to solve the construction of spanning trees and applied the algorithm to roadside-vehicle communication [20]. Da et al. proposed an improved maximum vertex cover algorithm to meet the strict time complexity constraint of mixed-integer linear programs (MILP), and multi-start local search handles it by combining the proposed algorithm with local search [21], more state-of-the-art is shown in the Table 1.

## 5. The Growth Optimizer Algorithm

### 5.1. Inspiration

Growth Optimization (GO) is a metaheuristic inspired by human behavior and how individuals develop in their surroundings [33]. In this metaheuristic, each potential solution is associated with an individual within a population. These individuals are ranked based on their Growth Resistance GR, which corresponds to the value of the objective function when evaluating the solution. This ranking divides the population into three groups based on a parameter $P_{1}$: the upper level (positions 1 to $P_{1}$, with the first being the leader), the middle level (positions $P_{1}$ + 1 to *N*−$P_{1}$), and the lower rank (positions *N*−$P_{1}$ to N).

### 5.2. Mathematical Modeling

The GO algorithm consists of two main phases for generating solutions: the learning phase and the reflection phase, as shown in Algorithm 1.
**Algorithm 1** The pseudocode of the GO algorithm.
**Input:** N(populationsize),D(populationdimension),ub,lb, $P_{1}=5$, $P_{2}=0.001$, $P_{3}=0.3$, FEs=0**Output:** Global optimal solution: $\vec{gbestx}$**1.** Initialize the population using $\vec{x}=lb+\left(ub-lb\right)\cdot \mathrm{rand}\left(N,D\right)$ and evaluate        (i=1,⋯,N)**while** FEs≤MaxFEs **do**    [∼,ind]=sort(GR)    $\vec{xbest}=\vec{x}\left(ind\left(1\right),:\right)$    **%Learning phase:**    **for** i=1 to *N* **do**        $\vec{x_{better}}=\vec{x}\left(ind\left(\mathrm{randi}\left(\left[2,1+P_{1}\right],1\right)\right),:\right)$        $\vec{x_{worse}}=\vec{x}\left(ind\left(\mathrm{randi}\left(\left[N-P_{1},N\right],1\right)\right),:\right)$        Find two random individuals that are different from ${\vec{x}}_{i}$: $\vec{x_{L1}}$ and $\vec{x_{L2}}$        Compute ${\mathrm{Gap}}_{k}$, k=1,2,3,4 by Equation (4)        Compute ${\mathrm{LF}}_{k}$, k=1,2,3,4 according to Equation (5)        Compute $SF_{i}$ according to Equation (6)        Compute ${\vec{KA}}_{k}$, k=1,2,3,4 according to Equation (8)        Complete the learning process for the *i*-th individual once according to Equation (7)        Complete the update of the *i*-th individual according to Equation (9)        Real-time update $\vec{gbestx}$        FEs=FEs+1    **end for**    **%Reflection phase:**    **for** i=1 to *N* **do**        Complete the reflection process for the *i*-th individual once according to Equations (10) and (11)        Complete the update of the *i*-th individual according to Equation (9)        Real-time update $\vec{gbestx}$        FEs=FEs+1    **end for****end while****Output:** $\vec{gbestx}$

#### 5.2.1. Learning Phase

In this phase, the algorithm generates movements by using the differences between individuals to reflect how much an individual should learn based on their knowledge gap compared to others, described in Equation (4)

(4) $$\begin{array}{c} \left\{\begin{array}{cc} {\vec{Gap}}_{1} & ={\vec{x}}_{best}-{\vec{x}}_{better}\\ {\vec{Gap}}_{2} & ={\vec{x}}_{best}-{\vec{x}}_{worse}\\ {\vec{Gap}}_{3} & ={\vec{x}}_{better}-{\vec{x}}_{worse}\\ {\vec{Gap}}_{4} & ={\vec{x}}_{L1}-{\vec{x}}_{L2} \end{array}\right. \end{array}$$

where ${\vec{x}}_{best}$ represents the best solution, while ${\vec{x}}_{better}$ represents one of the next $P_{1}-1$ best individuals. ${\vec{x}}_{worse}$ is one of the $P_{1}$ lowest ranked individuals in the population. Both ${\vec{x}}_{L1}$ and ${\vec{x}}_{L2}$ are random individuals different from the *i*th individual.

Metrics like learning factor $LF_{k}$ in Equation (5) and $SF_{i}$ in Equation (6) are used to control how much an individual should learn based on the knowledge gap and their resistance to change. $LF_{k}$ measures the influence of gap *k* on individual *i*, while $SF_{i}$ evaluates an individual’s resistance to change compared to the rest of the population.

(5) $$\begin{array}{cc} LF_{k} & =\frac{∥{\vec{Gap}}_{k}∥}{\sum_{k=1}^{4}∥{\vec{Gap}}_{k}\}},\;\left(k=1,2,3,4\right) \end{array}$$

(6) $$SF_{i}=\frac{GR_{i}}{GR_{max}}$$

To represent the acquired knowledge and generate a new candidate solution in Equation (7), the knowledge acquisition KA formula is used, allowing each individual *i* to absorb knowledge from various gaps using Equation (8)

(7) $${\vec{x}}_{i}^{It+1}={\vec{x}}_{i}^{It}+\vec{KA_{1}}+\vec{KA_{2}}+\vec{KA_{3}}+\vec{KA_{4}}$$

(8) $$\vec{KA_{k}}=SF_{i}\cdot LF_{k}\cdot {\vec{Gap}}_{k},\;\left(k=1,2,3,4\right)$$

where It is the number of current iterations, and ${\vec{x}}_{i}$ is the *i*th individual who absorbs the knowledge acquired.

Subsequently, an adjustment phase is carried out, where an evaluation is carried out of whether the solutions in the next iteration are better than the previous ones, modeled here as a minimization problem. If not, these solutions can be retained with a small retention probability, controlled by the parameters $P_{2}$ and $r_{1}$, a uniformly distributed random number in the range [0,1], preventing the loss of an individual’s effort, modeled as follows Equation (9):

(9) $${\vec{x}}_{i}^{It+1}=\left\{\begin{array}{cc} {\vec{x}}_{i}^{It+1} & \mathrm{if}\;f\left({\vec{x}}_{i}^{It+1}\right)<f\left({\vec{x}}_{i}^{It}\right)\\ \left\{\begin{array}{cc} {\vec{x}}_{i}^{It+1} & \mathrm{if}\;r_{1}<P_{2}\\ {\vec{x}}_{i}^{It} & \mathrm{else} \end{array}\right. & \mathrm{else} \end{array}\right.$$

#### 5.2.2. Reflection Phase

In this phase, individuals seek to compensate for or overcome their deficiencies. ub and lb are the upper and lower bounds of the search domain, $P_{3}$ is the parameter that controls the probability of reflection, and $r_{2}$, $r_{3}$, $r_{4}$, and $r_{5}$ are uniformly distributed random numbers in the range [0,1] used to determine how individuals adjust their solutions, modeled in Equations (10) and (11).

(10) $$x_{ij}^{t+1}=\left\{\begin{array}{cc} \left\{\begin{array}{cc} lb+r_{4}\cdot \left(ub-lb\right) & \mathrm{if}\;r_{3}<AF\\ x_{ij}^{It}+r_{5}\cdot \left(R_{j}-x_{ij}^{It}\right) & \mathrm{else}\; \end{array}\right. & \mathrm{if}\;r_{2}<P_{3}\\ x_{ij}^{It} & \mathrm{else} \end{array}\right.$$

(11) $$AF=0.01+0.99\times \left(1-\frac{FEs}{MaxFEs}\right)$$

The algorithm also incorporates an Attenuation Factor AF that depends on the current number of evaluations FE and the maximum number of evaluations *maxFE’s*. As the algorithm progresses, the AF value tends to converge to 0.001, indicating that individuals avoid frequent reinitialization and make the most of their progress. $\vec{R}$ denotes an individual at the high level, and it serves as a reflective learning guide for the current individual *i*. $R_{j}$ is the knowledge of the *j*th aspect of $\vec{R}$.

## 6. A New Binary Growth Optimizer

The binarization techniques used in continuous MHs involve transferring continuous domain values to binary domains, with the aim of maintaining the quality of moves and generating high-quality binary solutions. While some MHs operate on binary domains without a binary scheme, studies have demonstrated that continuous MHs supported by a binary scheme perform exceptionally well on multiple NP-hard combinatorial problems [34]. Examples of such MHs include the binary bat algorithm [35,36], binary particle swarm optimization [37], binary sine–cosine algorithm [38,39], binary salp swarm algorithm [40], binary grey wolf optimizer [39,41], binary dragonfly algorithm [42,43], binary whale optimization algorithm [39], and binary magnetic optimization algorithm [44]. In the scientific literature, two main groups of binary schemes used to solve combinatorial problems can be identified [45]. The first group refers to operators that do not cause alterations in the operations related to different elements of the MH. Within this group, two-step techniques stand out as the most widely used in recent years, as they are considered to be the most efficient in terms of convergence and their ability to find optimal solutions. These techniques are based on modifying the solution in the first step and discretizing it into a 0 or a 1 in the second step [34]. In addition, the angle modulation technique is also used in this group as it has been shown to be effective in solving combinatorial problems [46]. On the other hand, the second group of binary schemes includes methods that alter the normal operation of an MH. For example, the quantum binary approach, which is based on the application of quantum mechanisms to solve combinatorial problems [47]. In addition, also included in this group are set-based approaches, which focus on the selection of solution sets to improve the efficiency of the MH. Finally, clustering-based techniques, such as the k-means approach [48,49], are also considered in this second group, as they modify the normal operation of the MH to improve its ability to find optimal solutions.

**Figure 1:**
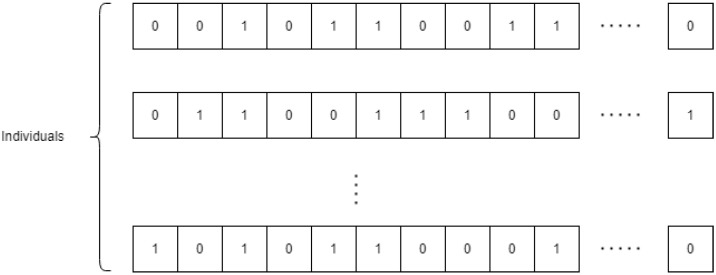
Solution representation.

Unlike the original approach of GO, where candidate solutions are found in the continuous domain, the proposed Binary Growth Optimizer (BWO) uses binary strings to represent solutions, where each decision variable corresponds to either 0 or 1, as shown in Figure 1.

This new proposal, detailed in pseudocode Algorithm 2, features the same moves as GO, with the addition of three key steps for obtaining binary solutions. First, the initialization of the initial population is generated in a binary manner, and binarization in two steps is carried out after the learning and reflection phases. These moves involve new parameters and functions in the algorithm that need to be adjusted as well.
**Algorithm 2** The pseudocode of the BGO algorithm.**Input:** $N\left(populationsize\right),D\left(populationdimension\right),ub,lb,P_{1}=5$, $P_{2}=0.001$, $P_{3}=0.3$, FEs=0**Output:** Global optimal solution: $\vec{gbestx}$**1.** Initialize the population using $\vec{x}=\mathrm{randint}\left(N,D\right)$ and evaluate (i=1,⋯,N)**while** FEs≤MaxFEs **do**    [∼,ind]=sort(GR)    $\vec{xbest}=\vec{x}\left(ind\left(1\right),:\right)$    **%Learning phase:**    **for** i=1 to *N* **do**        $\vec{x_{better}}=\vec{x}\left(ind\left(\mathrm{randi}\left(\left[2,1+P_{1}\right],1\right)\right),:\right)$        $\vec{x_{worse}}=\vec{x}\left(ind\left(\mathrm{randi}\left(\left[N-P_{1},N\right],1\right)\right),:\right)$        Find two random individuals that are different from ${\vec{x}}_{i}$: $\vec{x_{L1}}$ and $\vec{x_{L2}}$        Compute ${\mathrm{Gap}}_{k}$, k=1,2,3,4 by Equation (4)        Compute ${\mathrm{LF}}_{k}$, k=1,2,3,4 according to Equation (5)        Compute $SF_{i}$ according to Equation (6)        Compute ${\vec{KA}}_{k}$, k=1,2,3,4 according to Equation (8)        Complete the learning process for the *i*-th individual once according to Equation (7)        Complete the update of the *i*-th individual according to Equation (9)        Real-time update $\vec{gbestx}$        FEs=FEs+1    **end for**    **%Reflection phase:**    **for** i=1 to *N* **do**        Complete the reflection process for the *i*-th individual once according to Equations (10) and (11)        Complete the update of the *i*-th individual according to Equation (9)        Real-time update $\vec{gbestx}$        FEs=FEs+1    **end for**    **%Two-Step Binarization:**    **for** i=1 to *N* **do**        Compute the transfer function of the *i*-th individual        Complete the binarization of the *i*-th individual based on the binarization function selected    **end for****end while****Output:** $\vec{gbestx}$

### 6.1. Two-Step Binarization

In the scientific community, two-step binarization schemes are very relevant [50]. They have been widely used to solve a variety of combinatorial problems [51]. As the name suggests, this binarization scheme consists of two stages. The first stage involves the application of a transfer function [52], which transfers the values generated by the continuous MH to a continuous interval between 0 and 1. The second stage consists of the application of a binarization rule, which discretizes the numbers within that interval into binary values. This technique has been shown to be effective in solving combinatorial problems, since it allows the quality moves of the continuous MH to be preserved while generating high-quality binary solutions.

#### 6.1.1. Transfer Function

In 1997, Kennedy and his team [53] introduced the concept of transfer functions in the field of optimization. The significant advantage of these functions lies in their ability to provide probability values in a low computational cost range of 0 to 1. There are several types of transfer functions, with “S” and “V” forms being among the most popular [52,54]. Their utility is derived from their capacity to transform values generated by continuous metaheuristics into a continuous interval from 0 to 1.

It is important to note that there is no one-size-fits-all transfer function. This is due to the well-known “no free lunch” theorem [55], which states that there is no universal optimization algorithm that excels in all situations. Consequently, this theorem allows for experimentation and the development of new optimization algorithms.

#### 6.1.2. Binarization Rules

In the binarization stage, the conversion of discrete values into binary values, specifically 0 or 1, is obtained. Binarization rules are applied to the probabilities obtained through the transfer function to yield binary values. The choice of which binarization rule to use is crucial as it directly influences the effectiveness of the solutions in the binary metaheuristic context.

## 7. Results and Discussion

### 7.1. Experimental Setup

For the experiments, BWO was compared with three widely known metaheuristics, the grey wolf optimizer (GWO) [56], Pendulum Search Algorithm (PSA) [57], and sine–cosine algorithm (SCA) [58], and a selection of 49 different SCP instances was made. Each instance underwent a total of 31 experiments with V-type transfer function, the main features are shown in the Table 2, where the density corresponds to the percentage of non-zero in the matrix.

Regarding the configuration of the metaheuristics, we used the following strategies:

- Solution initialization: As a solution initialization strategy, we used the generation of random solutions. Since we were solving a binary problem, each decision variable was randomly assigned a value of 1 or 0.
- Termination conditions: In the literature, we found different term criteria, such as calls to the objective function [59] or the total number of iterations [60,61,62] that the metaheuristic will perform. We considered the total number of iterations as the completion criterion in our work. After previous experiments, it was determined to use 30 as the total number of iterations.
- Population size: As we worked with population metaheuristics, defining the number of individuals to use in the experimentation was key. After previous experiments, it was determined that 40 individuals would be used as the population size.

The hardware used in the experiments included an Intel Core i5-9400F processor operating at 2.90 GHz, 16.00 GB of RAM, and a 64-bit operating system with an x64 processor. Since we were experimenting with stochastic algorithms, we ran each experiment 31 times independently. All the code used for our experimentation can be found in [63].

These experiments were subjected to an analysis that delivered comparative convergence graphs, boxplots, time graphs, and exploration vs. exploitation charts. Additionally, a statistical analysis was conducted to assess the behavior of the Binary Growth Optimizer and enable a comparison with other implemented metaheuristics, thereby establishing a common framework for comparison with typical techniques.

### 7.2. Experimental Results

Table 3 shows the results obtained in the experimentation. Table 3 has the following format: The first column refers to the resolved SCP instance. The second column refers to the optimum of the instance. If the instance has no known optimum, it is marked with “-”. The third, fourth, and fifth columns repeat each metaheuristic used. The first of these three columns refers to the best result obtained in the 31 independent executions. The second of these three columns refers to the fitness average obtained in the 31 independent executions. The third of these three columns refers to the Relative Percentage Distance (RPD) calculated based on Equation (12),

(12) $$\mathrm{RPD}=\frac{100\cdot \left(Best-Opt\right)}{Opt}.$$

where Opt corresponds to the optimum of the instance and Best corresponds to the best value obtained for the experiment.

By reviewing Table 3, we can see that the BGO obtains competitive results compared to the other algorithms. We can even highlight that the BGO reaches the optimum in six instances.

### 7.3. Convergence vs. Iterations

**Figure 2:**
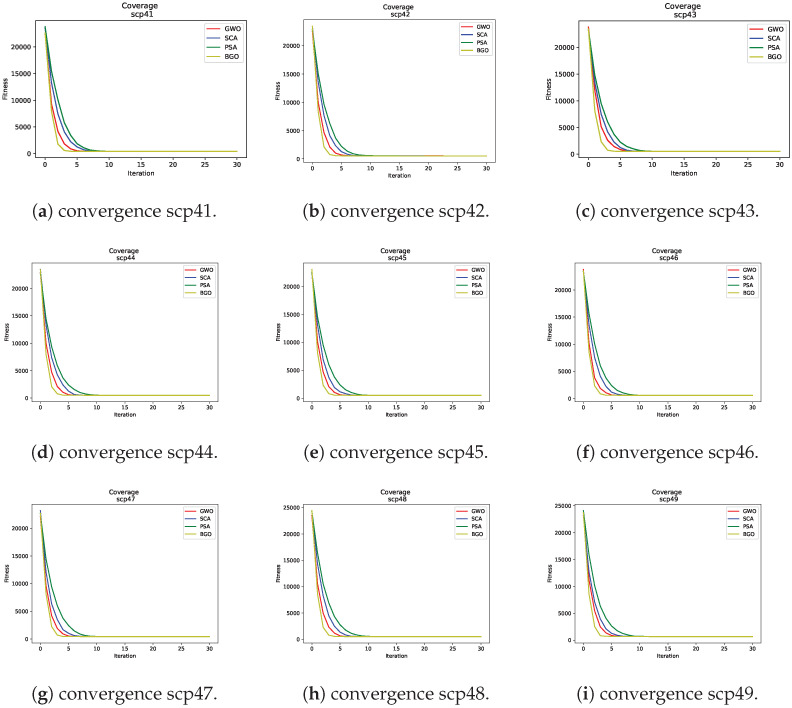

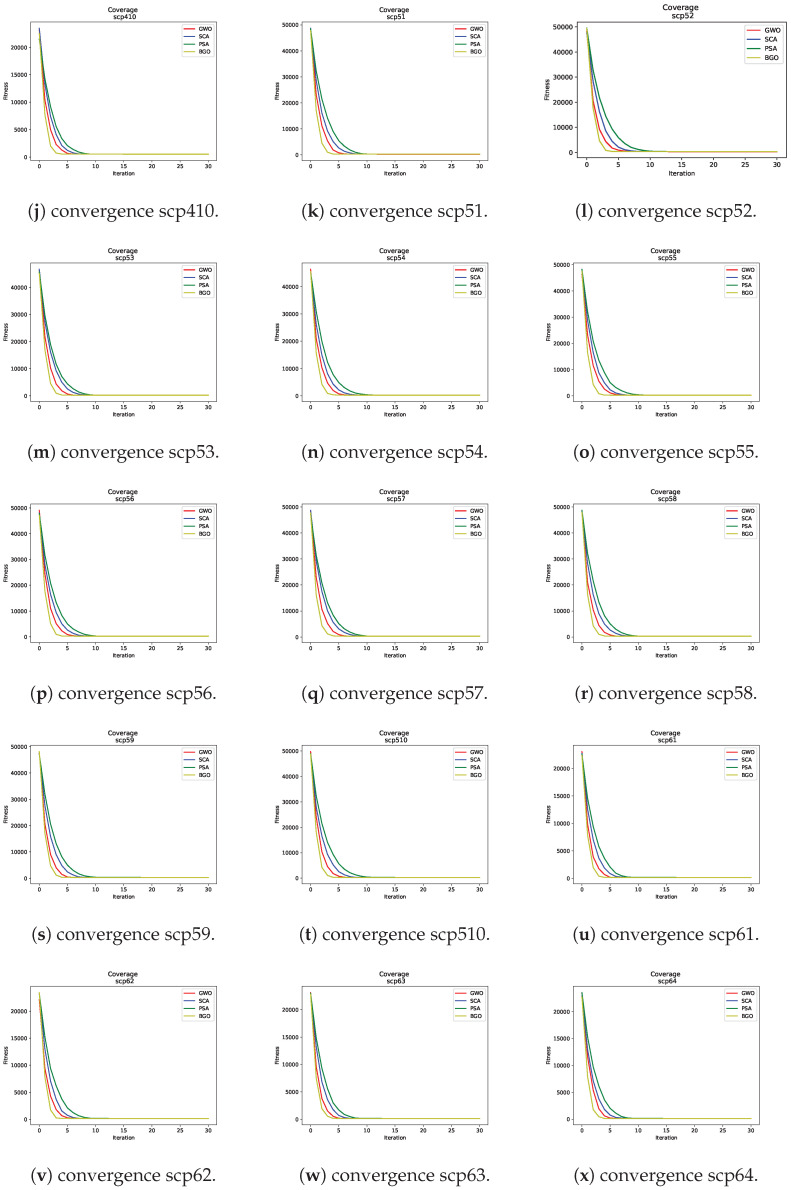

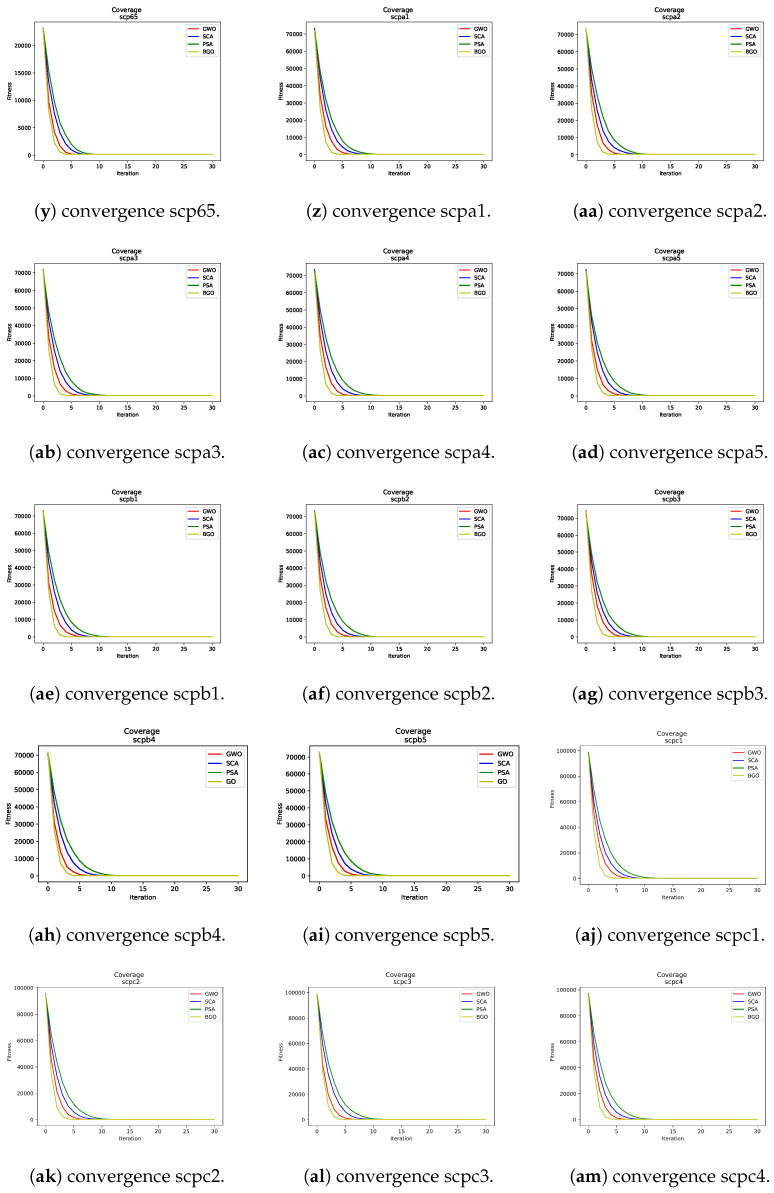

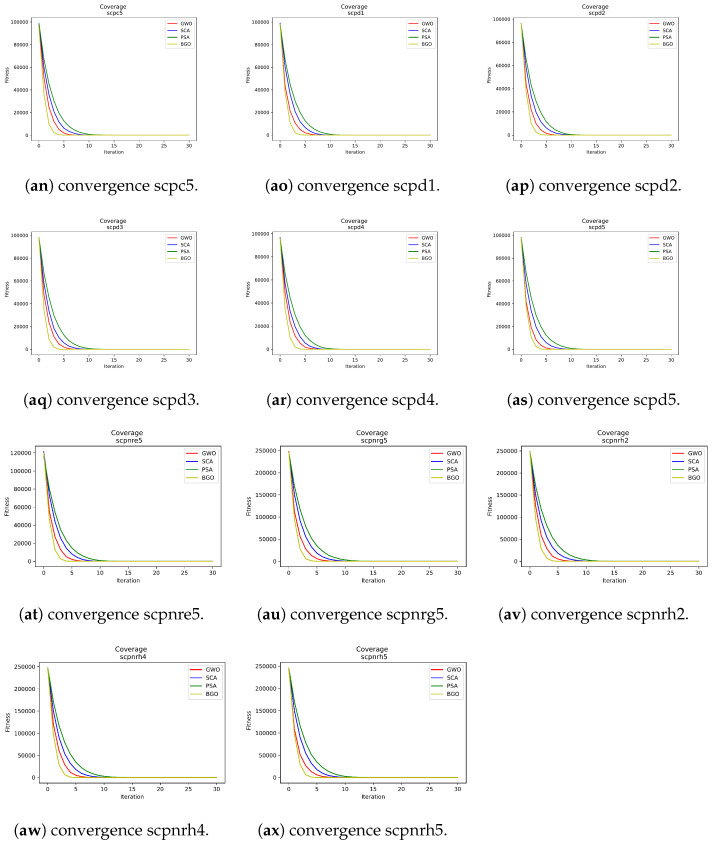
Fitness convergence curves.

One crucial aspect when evaluating the performance of metaheuristics is the speed at which they converge towards an optimal or near-optimal solution. In Figure 2, a consistent trend is observed: the Binary Growth Optimizer demonstrates a remarkable ability to converge more rapidly compared to the other three metaheuristics. Therefore, it can be highlighted for each of these:

- Binary Growth Optimizer: In all cases, the Binary Growth Optimizer exhibited faster convergence in fewer iterations. This suggests that this metaheuristic can find high-quality solutions in fewer iterations, which could be beneficial in practical applications where efficiency is crucial.
- Grey wolf optimizer: While the grey wolf optimizer did not reach the convergence speed of the Growth Optimizer, it still stood out as the second-best option in terms of convergence speed. This indicates its ability to find reasonable solutions within a reasonable time frame.
- Sine–cosine algorithm: This algorithm showed a moderate convergence speed compared to the two aforementioned metaheuristics. Although it may not be the fastest choice, it remains an effective metaheuristic for addressing SCP instances.
- Pendulum Search Algorithm: In the graphs, the PSA exhibited slower convergence compared to the other three metaheuristics. This suggests that it may require more iterations to reach comparable solutions.

### 7.4. Fitness Distribution

**Figure 3:**
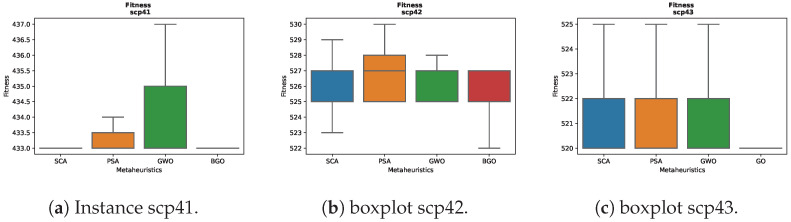

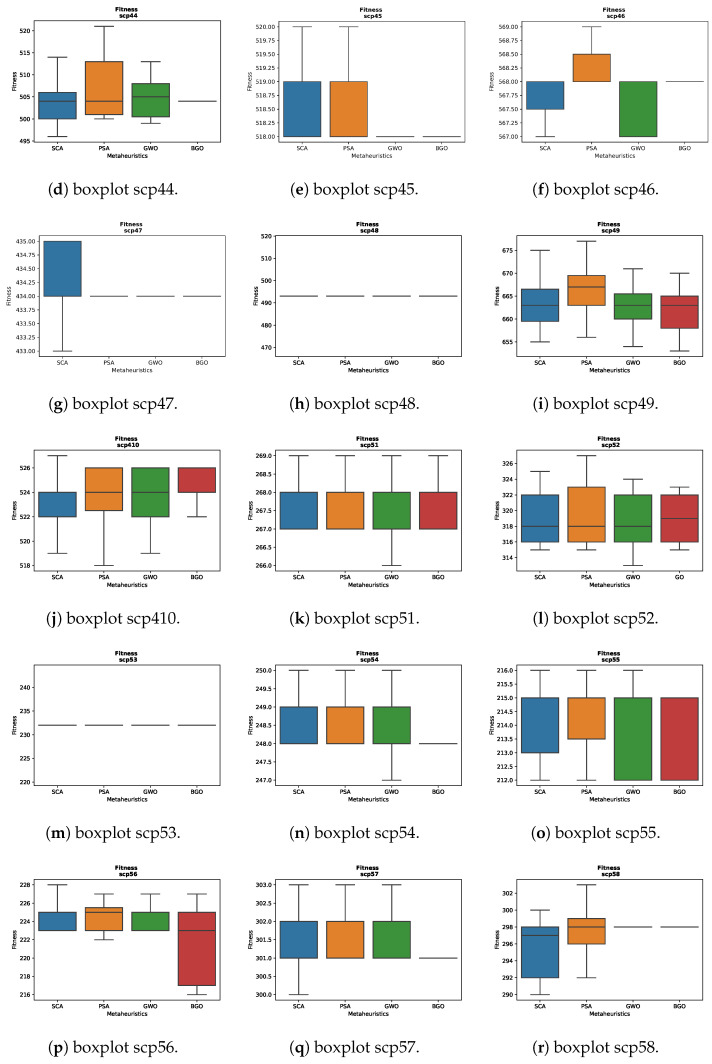

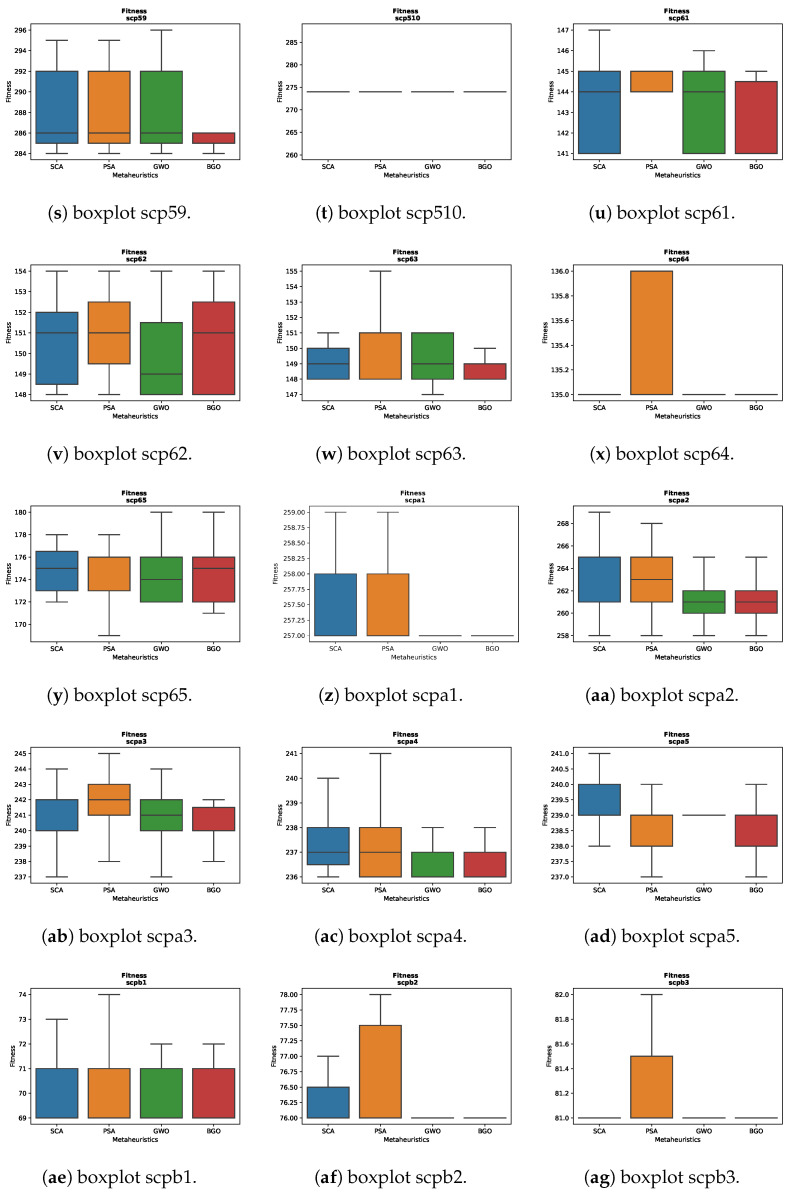

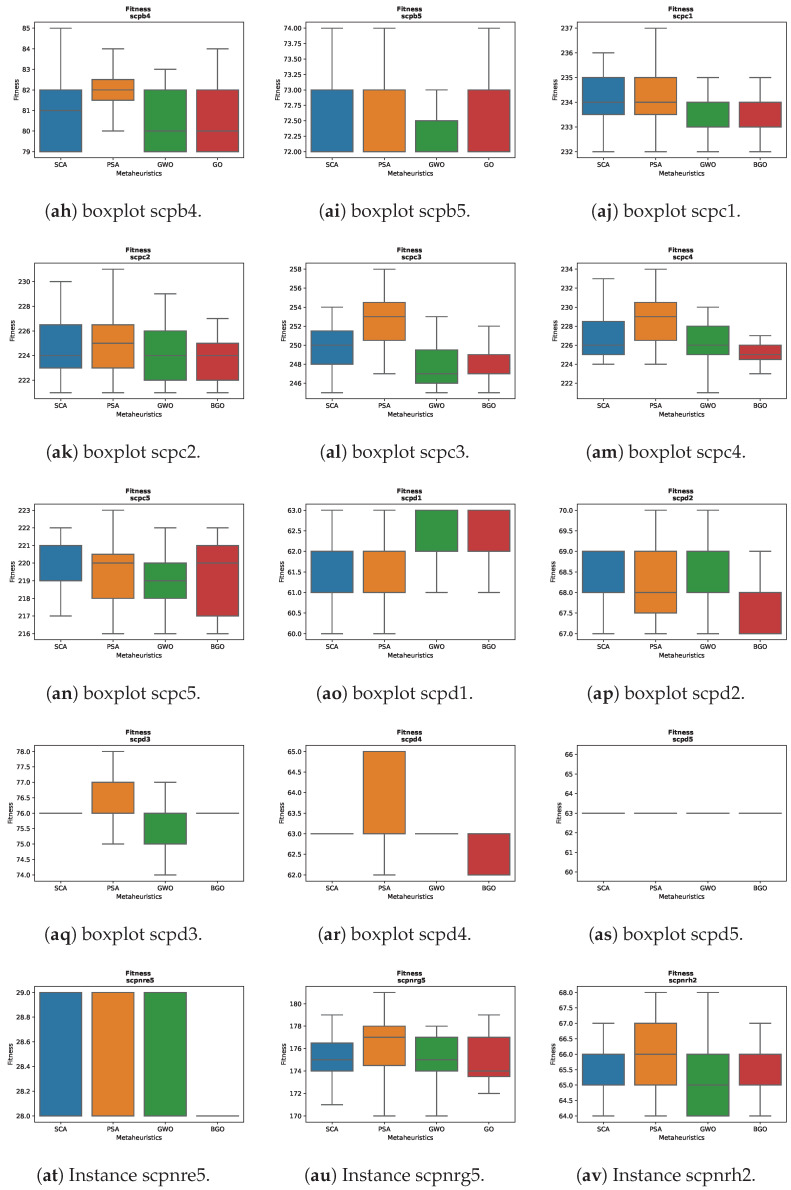

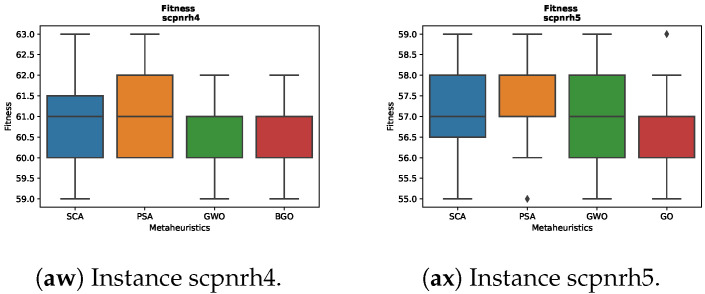
Boxplots.

For the instance scp43 in Figure 3c, significant differences in the results are observed among the various metaheuristics. The BGO displays a boxplot that is close to a line, indicating rapid convergence towards low fitness values. On the other hand, the other metaheuristics maintain more uniform boxplots, suggesting more consistent performance in this particular instance.

For the instance scp52 in Figure 3l, all the metaheuristics exhibit fairly similar performance, with minimal differences in the maximum and minimum values obtained. Particularly, the PSA presents higher maximum values compared to the others, while the GWO shows the lowest minimum values. This suggests that all the metaheuristics converge within a range of similar solutions in this instance, although the PSA tends to explore solutions with a higher maximum fitness value, indicating a broader search for high-quality solutions.

For the instance scpb4 in Figure 3ah, the results are mostly similar among the different metaheuristics, although it is observed that the PSA displays a slightly higher boxplot compared to the others. This suggests that in this particular instance, the PSA may have a tendency to explore solutions with a moderately higher level of fitness compared to the other metaheuristics.

For the instance scpb5 in Figure 3ai, an interesting pattern is observed in the results of the metaheuristics. All the metaheuristics show a distribution of fitness values that remains within a relatively narrow range. However, it is important to note that the PSA and GWO exhibit higher maximum fitness values compared to the other metaheuristics in this particular instance. This suggests that both the PSA and GWO have the capability to explore solutions with a higher maximum fitness value in this specific problem configuration.

This variation in the results emphasizes the importance of considering the characteristics and conditions of each individual instance when selecting the most suitable metaheuristic to address optimization problems.

For the instance scpnrg5 in Figure 3au, it is observed that all the metaheuristics maintain boxplots with high performance values. However, it is noteworthy that the BGO displays the lowest minimum values in this instance.

For the instance scpnrh2 in Figure 3av, it is observed that the SCA and BGO maintain similars boxplots, while the GWO suggests a tendency to converge towards lower fitness solutions while retaining the capability to reach higher values.

For the instance scpnrh4 in Figure 3aw, all the metaheuristics exhibit fairly similar performance, with minimal differences in the maximum and minimum values obtained.

For the instance scpnrh5 in Figure 3ax, interesting patterns are observed in the results of the different metaheuristics. In particular, the following are true:

- The SCA exhibits a complete range of maximum and minimum fitness values, suggesting a broad exploration of solutions.
- The PSA stands out for having higher maximum values than the other metaheuristics, although it also presents exceptionally low whiskers. This indicates its ability to reach high-quality solutions but also a tendency towards less optimal solutions.
- The GWO closely follows the SCA, covering a similar fitness range but with more lower solutions, indicating effective exploration of the search space.
- The BGO has values slightly lower than the average and shows whiskers at the upper end, suggesting a tendency to converge towards better optimal fitness solutions in this specific instance.

These results highlight how each metaheuristic has its own way of approaching the problem based on its specific characteristics and parameters, which can lead to varied results in different problem configurations.

### 7.5. Exploration vs. Exploitation

**Figure 4:**
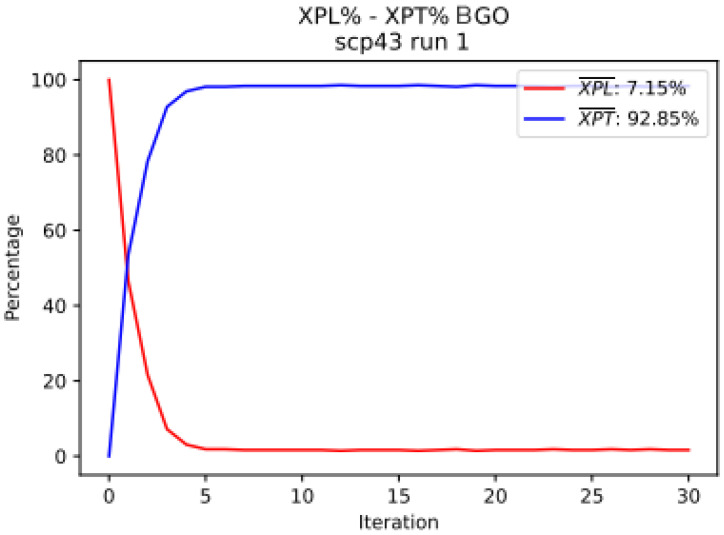
“Exploration vs. Exploitation” graph of the BGO metaheuristic when solving the scp43 instance.

Next, Figure 4 presents the exploration and exploitation of the BGO algorithm when solving the scp43 instance. This allows us to analyze the movements made by the metaheuristic and how it finds new solutions.

In the graph, quick convergence to 93 percent for exploitation and 7 percent for exploration is shown. This early convergence indicates that the metaheuristic is capable of finding a neighborhood of solutions close to the optimum in a few iterations.

### 7.6. Time vs. Iterations

**Figure 5:**
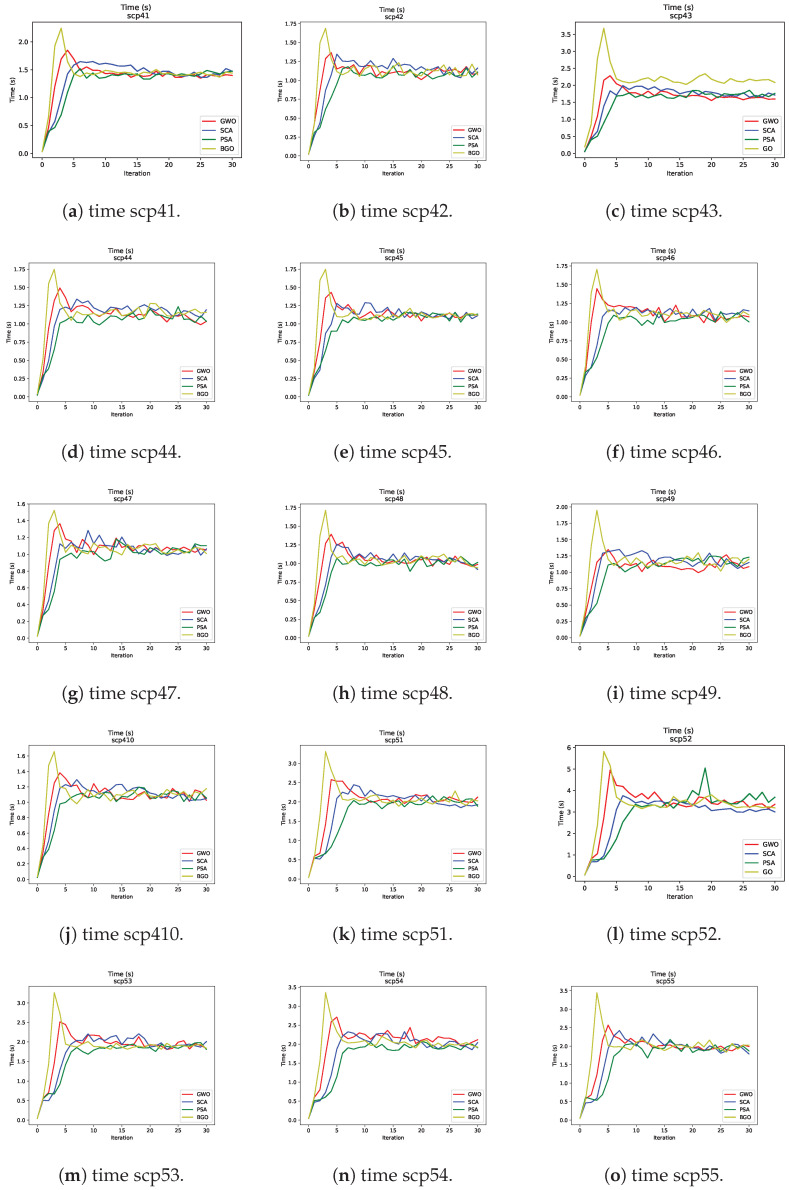

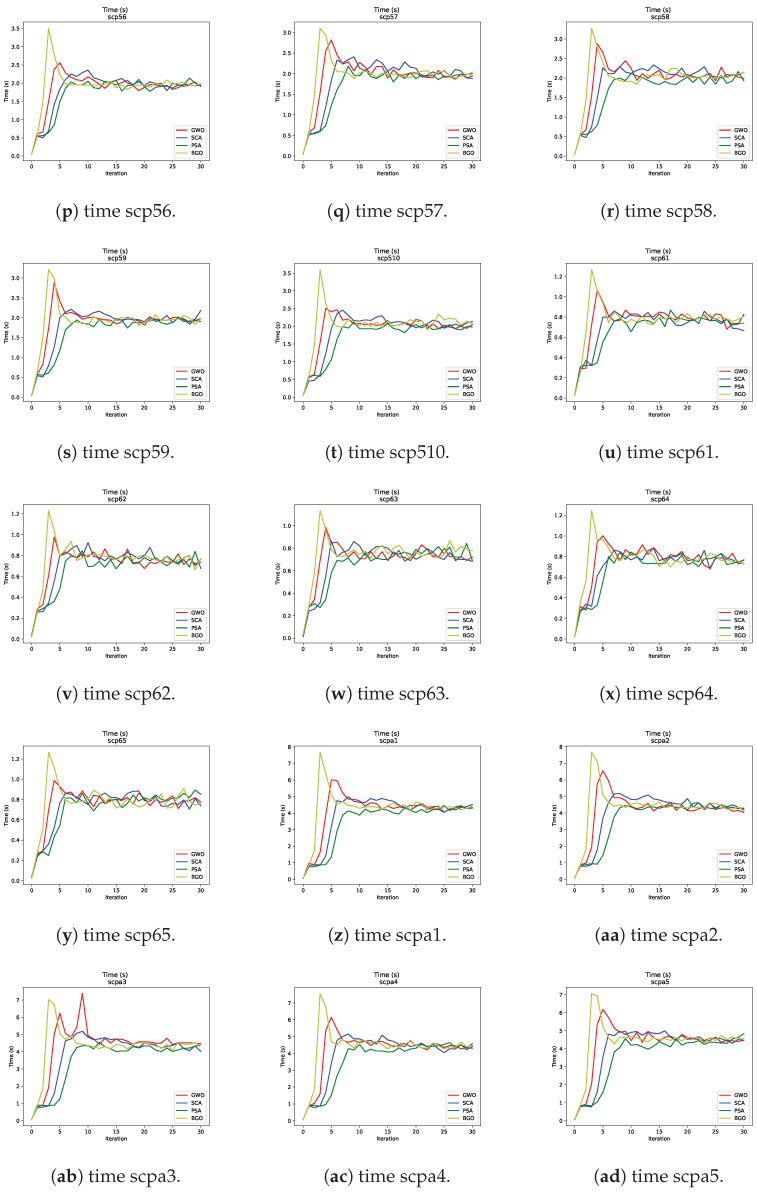

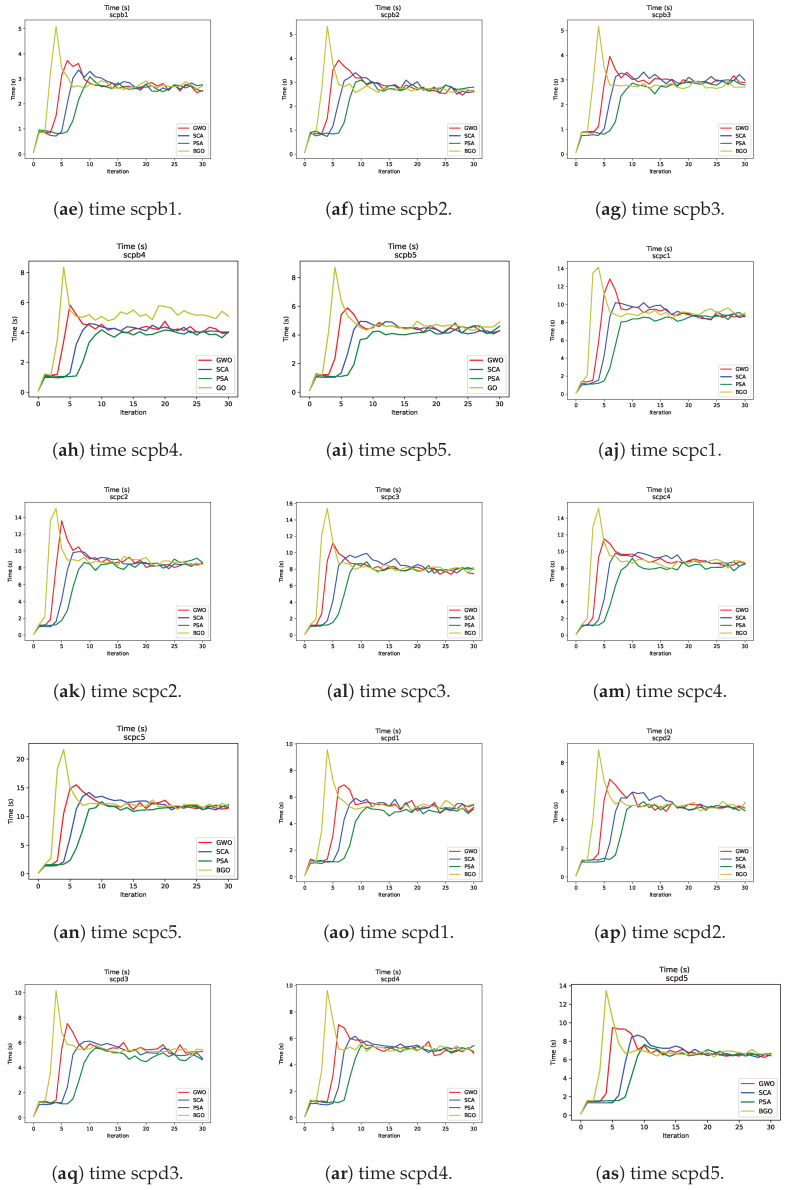

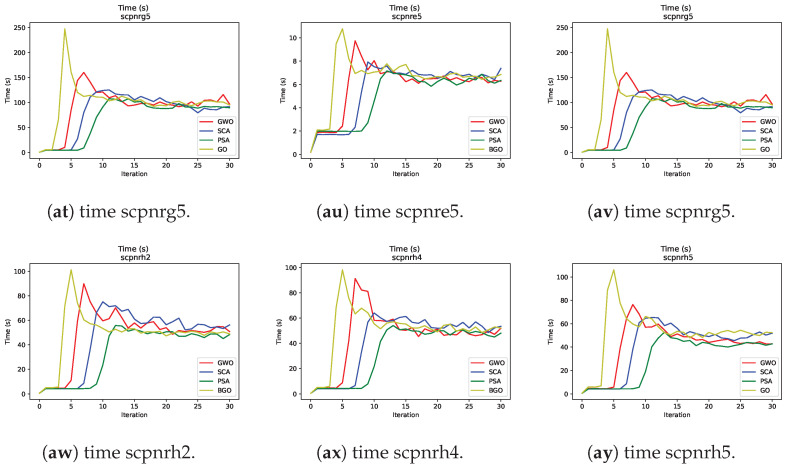
“Time vs. Iterations” graphs for the metaheuristics.

In Figure 5 are graphs that display time as a function of iterations for different metaheuristics in problem resolution. One notable finding is that the BGO requires more time in the initial iterations compared to the other metaheuristics.

This suggests that the BGO makes more time-consuming initial moves. However, this initial time investment has a significant benefit because the BGO tends to converge more rapidly towards high-quality solutions compared to the other metaheuristics. In other words, the BGO achieves early convergence towards an optimum in fewer iterations compared to the other MH.

### 7.7. Statistical Tests

Before conducting the statistical test, it was essential to determine the appropriate type of test to be employed, distinguishing between a parametric and a non-parametric test.

A non-parametric statistical test was deemed suitable for our analysis as the data originated from machines rather than exhibiting a normal distribution inherent in natural occurrences. Given the independence of our sample sets, the Wilcoxon–Mann–Whitney test [64,65] was selected for the statistical analysis. This test allows for the identification of algorithms that are significantly better than others. It offers detailed information about pairs of algorithms with significant differences in terms of performance.

Utilizing the scipy Python library, the chosen statistical test could be applied using the function scipy.stats.mannwhitneyu. Within this function, the “alternative” parameter was specified as “less”. The evaluation involved contrasting two distinct metaheuristics. The resulting *p*-value, less than 0.05, indicated that sample MhA was statistically smaller than sample MhB. Consequently, the following hypotheses were formulated in Equations (13) and (14):

(13) $$\begin{array}{cc} \; & H_{0}=\mathrm{MhA}\geq \mathrm{MhB} \end{array}$$

(14) $$\begin{array}{cc} \; & H_{1}=\mathrm{MhA}<\mathrm{MhB} \end{array}$$

In the event that the obtained *p*-value from the statistical test was less than 0.05, it could not be asserted that MhA exhibited inferior performance to MhB, leading to the rejection of $H_{0}$. This comparison was made in consideration of the problem being a minimization problem. To verify the findings presented, a Wilcoxon–Mann–Whitney test for each instance was conducted.

In Table 4, we can see the statistical test result when we compare BGO against the other three metaheuristics in each solved instance. When BGO is statistically better than another metaheuristic, we will indicate the *p*-value obtained in green and bold. When BGO is statistically worse than another metaheuristic, we will indicate the *p*-value obtained in red and in bold. In another case, there is no statistical difference between BGO and the other metaheuristic.

Based on the findings presented in Table 4, the summary in Table 5 illustrates the total occurrences where the BGO exhibits statistically lesser results (win) and statistically greater results (loss), and where there is no significant difference compared to the other metaheuristics.

Based on Table 5, the instances where the null hypothesis of the statistical test can be rejected (which states that there is no significant difference between the compared results) are are more clearly observable. It can be noted that in 61 instances, the BGO was statistically superior to its competitors, and in 85 instances, there was no statistically significant difference. Only in four instances did the obtained results favor the compared metaheuristic. These findings underscore the robust performance of the BGO, achieving strongly competitive and even superior outcomes.

The analysis of results across the twelve instances of the SCP reveals some interesting trends.

1. Rapid Convergence of the Binary Growth Optimizer (BGO): The BGO demonstrated swift convergence towards solutions with low fitness compared to the other metaheuristics. This suggests that the BGO can find high-quality solutions in fewer iterations.
2. High Competitiveness of BGO: Comparing the results obtained from Table 5, there is statistical evidence of the BGO’s performance compared to well-recognized metaheuristics, achieving very good and often superior results.
3. Behaviors across Different Instances: As expected, similar to its competitors, the BGO showcases varying behaviors across different studied instances, sometimes displaying highly precise fitness and sometimes not. It is crucial to consider this factor when implementing the BGO for problem-solving in real-world scenarios beyond controlled laboratory settings.
4. Success of Implementing Growth Optimizer with Binary Growth Optimizer: A Growth Optimizer is a high-performing metaheuristic in continuous optimization spaces, where various parameter configurations were tested to find the best-performing one. In this work, a Binary Growth Optimizer was employed using recommended parameters. However, altering these parameters might produce different, potentially superior outcomes.
5. Binarization Strategies: The use of binarization strategies is crucial for solving SCPs; utilizing the V-type transfer function yielded excellent results. Other transfer function types remain to be explored to observe the BGO’s behavior comprehensively.

In summary, each metaheuristic displayed its unique characteristics and strategies based on the specific instances of the SCP. The BGO stood out significantly for its rapid convergence in few iterations, high competitiveness, and excellent results.

### 7.8. Underlying Mechanisms

Next, the unique features of the BGO that facilitate such performance are shown, setting it apart from other metaheuristic methods.

- Integration of Learning and Reflection: The BGO distinguishes itself by its dual approach that combines two fundamental phases: the learning phase and the reflection phase. In the learning phase, the algorithm explores the search space by selecting “better” and “worse” solutions for each individual, fostering diversity and global exploration. On the other hand, in the reflection phase, the BGO employs reflection on decision variables to generate new random positions, promoting local exploitation and convergence towards optimal solutions.
- Adaptive Dynamics and Escape from Local Minima: The BGO introduces adaptive dynamics that dynamically adjust the probability of accepting worse solutions during the search. This feature allows the algorithm to adapt to the changing nature of the search landscape, increasing exploration in early stages and focusing on exploitation in later stages, thus enhancing the algorithm’s ability to find high-quality solutions in different types of optimization problems. To address the challenge of local minima, the BGO incorporates diversification mechanisms that enable the occasional acceptance of worse solutions with a small probability. This strategy helps the algorithm avoid getting stuck in suboptimal local minima and explore new regions of the search space, increasing the chances of finding globally optimal solutions.
- Well-defined Exploration and Exploitation Stages: The BGO is characterized by well-defined exploration and exploitation stages, allowing it to conduct extensive sampling of the search space in the early iterations and then focus on promising regions to refine solutions. This alternation between exploration and exploitation contributes to an efficient search and rapid convergence towards optimal solutions.
- Initialization with Longer Time and Rapid Convergence Compared to other metaheuristics: The BGO may require more time in initialization due to its dual approach and adaptive dynamics. However, once the search is underway, the BGO demonstrates faster convergence towards optimal solutions thanks to its ability to effectively explore and exploit the search space.

## 8. Conclusions

This analysis of twelve instances of the set-covering problem (SCP) revealed distinct trends among various metaheuristics. The Binary Growth Optimizer (BGO) emerged as a standout performer, showcasing rapid convergence towards solutions with low fitness in comparison to its counterparts. This rapid convergence suggests the BGO’s ability to attain high-quality solutions within a reduced number of iterations, signifying its efficiency in problem-solving.

The BGO’s success can be attributed to its unique mechanisms, including the integration of learning and reflection, adaptive dynamics, and well-defined exploration and exploitation stages. These mechanisms enable the BGO to adapt dynamically to changing landscapes, effectively explore diverse regions of the search space, and converge towards high-quality solutions in a reduced number of iterations. The BGO consistently achieved excellent and often superior results compared to established metaheuristics, showcasing its high competitiveness.

Moreover, statistical comparisons against well-established metaheuristics demonstrated the BGO’s high competitiveness, consistently achieving excellent and often superior results. However, it is essential to note that the BGO displayed diverse behaviors across different instances, implying variability in its performance. Understanding these variances is crucial when implementing the BGO for real-world problem-solving outside controlled environments.

The successful adaptation from the Growth Optimizer to the Binary Growth Optimizer highlights the potential for parameter optimization, indicating that fine-tuning parameters could potentially enhance the BGO’s performance further.

Additionally, the importance of binarization strategies in solving SCPs was underscored, particularly the success observed with the V-type transfer function. However, further exploration of alternative transfer function types remains an area for potential investigation to comprehensively understand the BGO’s behavior.

In essence, the BGO’s distinguishing traits of rapid convergence, competitiveness, and consistently excellent outcomes position it as a promising metaheuristic for solving SCPs while acknowledging the need for deeper exploration and parameter optimization for its maximal utilization. The unique approach of the BGO makes it suitable for a wide range of multidimensional optimization problems across various domains, including engineering, logistics, planning, and bioinformatics, given its ability to find high-quality solutions in complex search spaces. This versatility renders it a valuable tool for researchers and professionals facing optimization challenges in their respective fields.

## Figures and Tables

**Table 1:** Examples of metaheuristics used for set-covering problem resolutions and their application.

Ref.	Optimization Algorithm	Application	Convergence	Complexity
[22]	GA	Parameter calibration	Low	Low
[23]	Binary PSO	Input variable selection in ELM	Medium	High
[24]	Binary PSO	Parameter optimization in ELM	Medium	High
[25]	GA	Variable selection in hot metal desulfurization kinetics	Low	Low
[26]	Binary GWO	ESN	Low	High
[27]	Binary CSO	Parameter optimization in MRE isolator	Low	High
[28]	CSO and salp swarm algorithm	CNN	Medium	High
[29]	Binary CSA	CNN	Low	High
[30]	DE and binary DE	NN	Medium	High
[31]	Binary PSO and BBO	Set ret reduction	Fast	High
[32]	Binary PSO	Parameter optimization in Electric Vehicles	Medium	Low

**Table 2:** Description of instances.

Instance Set	Number of Instances	m	n	Cost Range	Density (%)	Optimal Solution
4	10	200	1000	[1, 100]	2	Known
5	10	200	2000	[1, 100]	2	Known
6	5	200	1000	[1, 100]	5	Known
A	5	300	3000	[1, 100]	2	Known
B	5	300	3000	[1, 100]	5	Known
C	5	400	4000	[1, 100]	2	Known
D	5	400	4000	[1, 100]	5	Known
NRE	5	500	5000	[1, 100]	10	Unknown
NRF	5	500	5000	[1, 100]	20	Unknown
NRG	5	1000	10,000	[1, 100]	2	Unknown
NRH	5	1000	10,000	[1, 100]	5	Unknown

**Table 3:** Results obtained.

		SCA			PSA			GWO			BGO		
**Inst.**	**Opt.**	**Min**	**Avg**	**RPD**	**Min**	**Avg**	**RPD**	**Min**	**Avg**	**RPD**	**Min**	**Avg**	**RPD**
41	429	431.0	433.75	0.466	431.0	433.7812	0.4662	433.0	434.0	0.9324	433.0	433.03125	0.9324
42	512	523	527	2.1484	517	528.2903	0.9766	525	526.2258	2.5391	518	525.7097	1.1719
43	516	520	521.0625	0.7752	520	521.4688	0.7752	520	520.8750	0.7752	520	520.4063	0.7752
44	494	496	504.4516	0.4049	500	506.5806	1.2146	499	505.4194	1.0121	499	504.4839	1.0121
45	512	514	518.2903	0.3906	518	519.6774	1.1719	518	518.1290	1.1719	518	518	1.1719
46	560	564	567.8065	0.7143	565	569	0.8929	564	567.7742	0.7143	567	567.8710	1.2500
47	430	432	434.2903	0.4651	433	434.2581	0.6977	432	434	0.4651	433	433.9677	0.6977
48	492	493	494.0645	0.2033	493	494.3871	0.2033	493	493.8387	0.2033	493	493.2903	0.2033
49	641	655	663.7742	2.1841	656	667.5161	2.3401	654	662.7742	2.0281	653	662.0968	1.8721
410	514	517	522.6774	0.5837	518	523.6129	0.7782	519	523.4194	0.9728	517	524.0645	0.5837
51	253	267	267.8387	5.5336	267	267.7742	5.5336	257	267.0323	1.5810	267	267.4839	5.5336
52	302	315	318.6563	4.3046	315	319.5	4.3046	313	319.125	3.6424	315	319.0938	4.3046
53	226	229	231.9032	1.3274	230	232.0323	1.7699	229	231.8387	1.3274	232	232	2.6549
54	242	244	247.8387	0.8264	244	248.3226	0.8264	244	247.9032	0.8264	244	248.0968	0.8264
55	211	212	213.5161	0.4739	212	214.4516	0.4739	212	213.4194	0.4739	212	213.0645	0.4739
56	213	216	223.1613	1.4085	216	223.3548	1.4085	216	223.3548	1.4085	216	221.9032	1.4085
57	293	299	301.6774	2.0478	296	302.1935	1.0239	297	301.8065	1.3652	299	301.2903	2.0478
58	288	290	295.7097	0.6944	290	297.5161	0.6944	290	297.6129	0.6944	290	297.3871	0.6944
59	279	284	287.5161	1.7921	284	288.1290	1.7921	284	288.2581	1.7921	284	286.2258	1.7921
510	265	272	274.1935	2.6415	272	274.4194	2.6415	272	273.8710	2.6415	273	274.0323	3.0189
61	138	141	143.3871	2.1739	141	144.0968	2.1739	141	143.0323	2.1739	141	142.2258	2.1739
62	146	148	150.4839	1.3699	148	151.0645	1.3699	148	150.0968	1.3699	148	150.4194	1.3699
63	145	148	149.2258	2.0690	148	150.0323	2.0690	147	149.2258	1.3793	148	148.6129	2.0690
64	131	134	135.3871	2.2901	135	135.3871	3.0534	135	135.1290	3.0534	134	135.2258	2.2901
65	161	172	174.8710	6.8323	165	174.8065	2.4845	172	174.5161	6.8323	171	174.4839	6.2112
a1	253	257	257.5484	1.5810	257	257.6774	1.5810	257	257	1.5810	257	257.0645	1.5810
a2	252	258	262.2581	2.3810	258	263.1290	2.3810	258	261.3871	2.3810	258	261.1290	2.3810
a3	232	235	241	1.2931	236	241.7742	1.7241	237	240.8387	2.1552	235	240.3226	1.2931
a4	234	236	237.4839	0.8547	236	237.0645	0.8547	236	236.7419	0.8547	236	236.6129	0.8547
a5	236	237	239.3226	0.4237	237	238.6774	0.4237	237	238.7742	0.4237	237	238.4516	0.4237
b1	69	69	70.4839	0	69	70.6452	0	69	70.2581	0	69	70.2258	0
b2	76	76	76.5806	0	76	77.1613	0	76	76.3548	0	76	76.1935	0
b3	80	80	81.2258	0	80	81.2581	0	80	81.1290	0	81	81.1613	1.2500
b4	79	79	81.0968	0	79	81.8065	0	79	80.5806	0	79	80.5161	0
b5	72	72	72.3871	0	72	72.5484	0	72	72.2903	0	72	72.5484	0
c1	227	232	234.0968	2.2026	231	234.3548	1.7621	232	233.5161	2.2026	232	233.4194	2.2026
c2	219	221	224.5161	0.9132	221	225	0.9132	221	224.1613	0.9132	221	223.7419	0.9132
c3	243	245	249.7742	0.8230	247	252.2581	1.6461	245	248.0323	0.8230	245	247.7742	0.8230
c4	219	224	226.9677	2.2831	224	228.8387	2.2831	221	226.5806	0.9132	222	225.5161	1.3699
c5	215	217	219.6129	0.9302	216	219.5161	0.4651	216	218.8387	0.4651	216	219.0645	0.4651
d1	60	60	61.9355	0	60	61.8065	0	60	62.1290	0	61	62.1613	1.6667
d2	66	67	68.2258	1.5152	67	68.0968	1.5152	67	68.1290	1.5152	67	67.7742	1.5152
d3	72	73	75.8065	1.3889	73	76.2903	1.3889	74	75.6774	2.7778	74	75.8387	2.7778
d4	62	62	63.0968	0	62	63.6774	0	62	63.2581	0	62	62.8387	0
d5	61	63	63.1613	3.2787	62	63.2903	1.6393	63	63.1613	3.2787	63	63.0323	3.2787
nre5	-	28	28.2813	-	28	28.5313	-	28	28.3125	-	28	28.1563	-
nrg5	-	171	175.3125	-	168	176.3125	-	170	175.0313	-	172	175.0625	-
nrh2	-	64	65.7419	-	64	66.0323	-	64	65.3226	-	64	65.2581	-
nrh4	-	59	60.9355	-	60	61.1935	-	59	60.4516	-	59	60.3548	-
nrh5	-	55	57.1613	-	55	57.5161	-	55	57	-	55	56.5484	-

**Table 4:** Matrix of *p*-values from the Wilcoxon–Mann–Whitney test for the BGO against the SCA, PSA, and GWO in different instances. In green the *p*-values lower than 0.05 where BGO exhibits statistically lesser results, and in red the *p*-values greater than 0.95 where BGO exhibits statistically greater results compared to other metaheuristics.

	SCA	PSA	GWO
41	**0.032526**	**0.017652**	**0.00505**
42	0.083416	**0.008453**	0.178185
43	0.142326	**0.017925**	**0.040147**
44	0.673895	0.358608	0.17129
45	**0.000777**	**0.000075**	**0.020949**
46	0.777121	0.101089	0.947336
47	**0.026426**	**0.027054**	0.221067
48	**0.049813**	**0.015255**	0.092276
49	0.231335	**0.000373**	0.457734
410	**0.971242**	0.800105	0.837222
51	0.08422	0.204932	0.769852
52	0.656474	0.235486	0.368845
53	0.849024	0.509173	0.926297
54	0.378881	**0.037574**	0.588779
55	**0.020766**	**0.000228**	0.174886
56	0.194771	0.069321	0.212655
57	**0.008287**	**0.006762**	**0.049149**
58	**0.990298**	0.424729	0.538867
59	**0.025665**	**0.003579**	**0.027499**
510	0.379496	0.110218	0.883339
61	**0.013689**	**0.00103**	0.052988
62	0.396878	0.125416	0.666065
63	0.115385	0.09021	0.154446
64	0.297105	**0.034655**	0.552952
65	0.185046	0.147401	0.482672
a1	**0.000253**	**0.008388**	0.926324
a2	0.124053	**0.002265**	0.431456
a3	**0.016308**	**0.000256**	0.100149
a4	**0.003371**	**0.012922**	0.550884
a5	**0.000524**	0.082315	**0.023963**
b1	0.213058	0.151325	0.43297
b2	0.146666	**0.038143**	0.375167
b3	0.345372	0.245959	0.612847
b4	0.090897	**0.002352**	0.33707
b5	0.887149	0.548425	**0.975947**
c1	**0.002907**	**0.001879**	0.385972
c2	0.114905	**0.028943**	0.175642
c3	**0.000398**	**0.0**	0.539888
c4	**0.002055**	**0.000006**	**0.039399**
c5	**0.026575**	0.320787	**0.991447**
d1	0.892595	0.950455	0.506028
d2	**0.010529**	0.06074	**0.033358**
d3	0.31389	**0.004401**	0.887789
d4	**0.039419**	**0.000385**	**0.025442**
d5	0.504675	0.097037	0.250647
nre5	0.1169	**0.000888**	0.072876
nrg5	0.202966	**0.009097**	0.41873
nrh2	**0.013674**	**0.003689**	0.440986
nrh4	**0.004821**	**0.000504**	0.335568
nrh5	**0.011639**	**0.000306**	0.052988

**Table 5:** Matrix of ocurrences from Wilcoxon–Mann–Whitney tests.

Instance	Win	No Significant Difference	Loss
41	3	0	0
42	1	2	0
43	2	1	0
44	0	3	0
45	3	0	0
46	0	3	0
47	2	1	0
48	2	1	0
49	1	2	0
410	0	2	1
51	0	3	0
52	0	3	0
53	0	3	0
54	1	2	0
55	2	1	0
56	0	3	0
57	3	0	0
58	0	2	1
59	3	0	0
510	0	3	0
61	2	1	0
62	0	3	0
63	0	3	0
64	1	2	0
65	0	3	0
a1	2	1	0
a2	1	2	0
a3	2	1	0
a4	2	1	0
a5	2	1	0
b1	0	3	0
b2	1	2	0
b3	0	3	0
b4	1	2	0
b5	0	2	1
c1	2	1	0
c2	1	2	0
c3	2	1	0
c4	3	0	0
c5	1	1	1
d1	0	3	0
d2	2	1	0
d3	1	2	0
d4	3	0	0
d5	1	2	0
nre5	1	2	0
nrg5	1	2	0
nrh2	2	1	0
nrh4	2	1	0
nrh5	2	1	0
Total	61	85	4

## Data Availability

All the results of this work are available at the GitHub repository (https://github.com/BenjaminAleRamosT/BGO/tree/main (accessed on 30 April 2024)).

## Abbreviations

The following abbreviations are used in this manuscript:

MH	Metaheuristic
SCP	Set-covering problem
P	Polynomial time
NP	Nondeterministic polynomial time
EAs	Evolutionary algorithms
MSA	Binary monkey search algorithm
ALO	Antlion optimization
CSA	Crow search algorithm
GA	Genethic Algorithm
MLST	Minimum labeling spanning tree
VANETs	Vehicular ad hoc networks
MST	Minimum spanning tree
MILP	Mixed-integer linear programs
ESN	Echo state network
ELM	Extreme Learning Machines
MRE	Magnetorheological elastomer
CNN	Convolutional neural network
NN	Neural network
DE	Differential evolution
GO	Growth optimizer
BGO	Binary Growth Optimizer
GR	Growth resistance
$I_{t}$	Current iteration
LF	Learning factor
KA	Knowledge acquisition
AF	Attenuation factor
FE	Current number of evaluations
MaxFE	Maximum number of evaluations
ub	Domain upper bound
lb	Domain lower bound
D	Population dimension
GWO	Grey wolf optimizer
PSA	Pendulum Search Algorithm
SCA	Sine–cosine algorithm
